# IMPROVE*job* – Participatory intervention to improve job satisfaction of general practice teams: a model for structural and behavioural prevention in small and medium-sized enterprises – a study protocol of a cluster-randomised controlled trial

**DOI:** 10.1186/s13063-020-04427-7

**Published:** 2020-06-16

**Authors:** Birgitta M. Weltermann, Christine Kersting, Claudia Pieper, Tanja Seifried-Dübon, Annegret Dreher, Karen Linden, Esther Rind, Claudia Ose, Karl-Heinz Jöckel, Florian Junne, Brigitte Werners, Verena Schroeder, Jean-Marie Bois, Achim Siegel, Anika Thielmann, Monika A. Rieger, Stefanie Kasten, on behalf of the IMPROVEjob consortium, M. A. Rieger, E. Rind, A. Siegel, A. Wagner, E. Tsarouha, B. Weltermann, S. Kasten, K. Linden, L. Degen, A. Thielmann, F. Junne, T. Seifried-Dübon, A. Hermann-Werners, F. Stuber, S. Zipfel, B. Werners, M. Grot, K-H Jöckel, C. Pieper, V. Schröder, J-M Bois, A-L Eilerts, M. Brinkmann, C. Kersting, S. Emerich, S. Burgess, M. Hippler, A. Dreher, C. Ose, L. Koppka, J. Block

**Affiliations:** 1 Institute of General Practice and Family Medicine, University Hospital Bonn, University of Bonn, Venusberg-Campus 1, 53127 Bonn, Germany; 2 Institute for General Medicine, University Hospital Essen, University of Duisburg-Essen, Hufelandstr 55, 45122 Essen, Germany; 3 Institute for Medical Informatics, Biometry and Epidemiology, University Hospital Essen, University of Duisburg-Essen, Hufelandstr 55, 45147 Essen, Germany; 4 Department of Psychosomatic Medicine and Psychotherapy, University Hospital Tuebingen, Osianderstraße 5, 72076 Tuebingen, Germany; 5 Institute of Occupational and Social Medicine and Health Services Research, University Hospital Tuebingen, Wilhelmstr 27, 72074 Tuebingen, Germany; 6 Center for Clinical Trials, University Hospital Essen, University of Duisburg-Essen, Hufelandstr. 55, 45147 Essen, Germany; 7 Institute for Operations Research, Ruhr University Bochum, Universitätsstr 150, 44801 Bochum, Germany

**Keywords:** Job satisfaction, Perceived psychological stress, Primary care, General practices, Participatory intervention, Psychological well-being, Effectiveness, Leadership, Structural prevention, Behavioural prevention

## Abstract

**Background:**

Perceived high chronic stress is twice as prevalent among German general practitioners (GPs) and non-physician medical staff compared to the general population. The reasons are multi-factorial and include patient, practice, healthcare system and societal factors, such as multi-morbidity, the diversity of populations and innovations in medical care. Also, practice-related factors, like stressful patient-staff interactions, poor process management of waiting times and lack of leadership, play a role. This publicly funded study evaluates the effectiveness of the newly developed participatory, interdisciplinary, and multimodal IMPROVE*job* intervention on improving job satisfaction among general practice personnel. The intervention aims at structural stress prevention with regard to working conditions and behavioural stress prevention for leaders and other practice personnel.

**Methods:**

In this cluster-randomised controlled trial, a total of 56 general practices will be assigned to either (1) participation in the IMPROVE*job* intervention or (2) the waiting-list control group. The IMPROVE*job* intervention consists of the following elements: three workshops, a toolbox with supplemental material and an implementation period with regular contact to so-called IMPROVE*job* facilitators. The first workshop, addressing leadership issues, is designed for physicians with leadership responsibilities only. The two subsequent workshops target all GP and non-physician personnel; they address issues of communication (with patients and within the team), self-care and team-care and practice organisation. During the 9-month implementation period, practices will be contacted by IMPROVE*job* facilitators to enhance motivation. Additionally, the practices will have access to the toolbox materials online. All participants will complete questionnaires at baseline and follow up. The primary outcome is the change in job satisfaction as measured by the respective scale of the validated German version of the Copenhagen Psychosocial Questionnaire (COPSOQ, version 2018). Secondary outcomes obtained by questionnaires and - qualitatively - by facilitators comprise psychosocial working conditions including leadership aspects, expectations and experiences of the workshops, team and individual efforts and organisational changes.

**Discussion:**

It is hypothesised that participation in the IMPROVE*job* intervention will improve job satisfaction and thus constitute a structural and behavioural prevention strategy for the promotion of psychological wellbeing of personnel in general practices and prospectively in other small and medium sized enterprises.

**Trial registration:**

German Clinical Trials Register: DRKS00012677. Registered on 16 October 2019. Retrospectively, https://www.drks.de/drks_web/navigate.do?navigationId=trial. HTML&TRIAL_ID = DRKS00012677.

## Contributions to the literature

This study will contribute as follows:

1. Few studies have evaluated participatory intervention strategies to improve job satisfaction in small and medium sized enterprises
2. This study protocol describes a cluster randomised trial aiming to improve job satisfaction in general practice personnel
3. The intervention aims at activating the target group
4. The study comprises behavioural and structural prevention strategies
5. Issues of leadership, communication and work organisation in practices are addressed.

## Background

Poor job satisfaction is an ongoing issue across many job domains worldwide [1, 2]. Due to the shortage of physicians in primary care in many countries, increased research efforts are directed to how to maintain and improve job satisfaction in this workforce [3–5]. Studies in various general practice populations in Europe and beyond showed that job satisfaction is profoundly influenced by work-related factors [5–7]. Factors that are known to decrease job satisfaction include too many working hours, administrative burdens, inadequate income, heavy workload, and lack of time and recognition [7]. Persistently low job satisfaction is related not only to chronic stress, burnout, depression, early retirement and other indicators of physicians’ health, but is also linked to less satisfactory patient outcomes [8–15]. A study by Viehmann et al. showed that physicians and non-physician personnel in general practices are twice as likely to experience high chronic stress compared to the general population [10]. In the same population, physicians in group practices had a higher rate of burnout than those from single practices, with young, part-time, female physicians employed there having the highest burnout rate, even when compared to group practice owners [16]. Various reasons for high strain were identified; key factors were quality of leadership, difficult patient-encounters and practice organisation [10, 17, 18].

Various approaches to improving the mental wellbeing of healthcare workers have been evaluated [19, 20]. Many approaches address individual behaviour such as stress management, meditation, or training in self-care [21, 22]. However, based on the European principles of occupational health and safety, interventions should first target the work environment of individuals and address behavioural prevention thereafter [23]. A review by Montano et al. about the effectiveness of organisation-related interventions showed that health-promoting effects were achieved among employees when interventions simultaneously focused on work equipment, work processes and working conditions [24].

Having identified lack of leadership, difficult patient-encounters and practice organisation as problems in German general practices [10, 18], the IMPROVE*job* intervention was developed under the leadership of a family medicine physician and scientist, following an interdisciplinary and participatory approach: experts from the fields of general practice and family medicine, occupational and psychosomatic medicine, operations research, health promotion and epidemiology provided input into the various areas; a research support group with GPs and practice assistants was repeatedly asked for input and feedback to ensure that the intervention is tailored to the needs and capacities of general practice teams. The intervention focuses on reducing work-related psychological stress, to increase job satisfaction. It consists of three workshops (i.e. a leadership workshop for physicians with leadership responsibilities and two workshops for the entire practice team), a toolbox and so-called IMPROVE*job* facilitators to support implementation. Learning contents for participants focus on issues relating to leadership, communication and work processes. The main aim of this cluster-randomised controlled trial (cRCT) is to investigate whether the IMPROVE*job* intervention increases job satisfaction amongst general practice personnel.

## Methods/design

### Study design

**Fig. 1:**
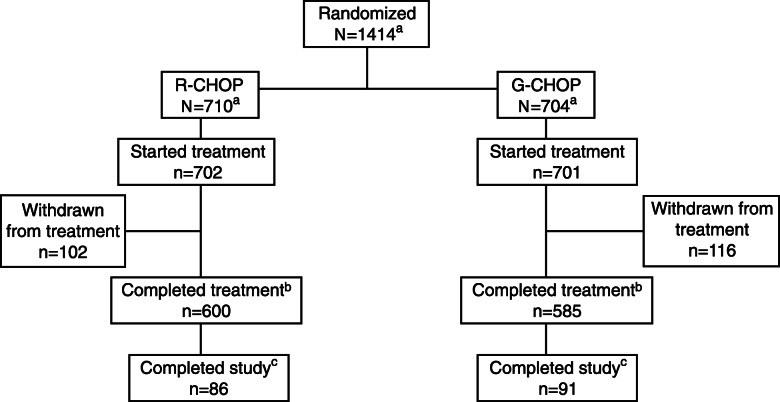
Study design: cluster-randomised controlled trial with intervention practices and waiting-list control practices

This cRCT evaluates the effectiveness of the IMPROVE*job* intervention in improving job satisfaction (primary outcome) among practice personnel. Randomisation will take place at practice level, i.e. all personnel in the practice will be assigned either to the intervention or to the control group. The study will be conducted according to the waiting-list control principle, i.e. practices of the intervention group will receive the IMPROVE*job* intervention after the baseline data collection; the control group will receive the workshops after completion of the study. For details see Fig. 1.

### Participants and recruitment

The study will be conducted in general practices of the North Rhine region in Germany. Urban and rural medical practices are selected to ensure better generalisation of the results. Practices will be drawn as random samples from an official list of registered general practice physicians of the Association of Statutory Health Insurance Physicians of North Rhine (in German, *Kassenärztliche Vereinigung Nordrhein*) in these selected areas and from a list of teaching practices of regional universities. We previously showed that teaching practices are typically easier to recruit and do not differ from non-teaching practices with regard to health service characteristics [25]. We therefore aimed for an approximately equal distribution of teaching and non-teaching practices in the final sample.

The practices will be contacted by the Institute of General Practice and Family Medicine of the University of Bonn. Depending on available contact details, practices will be sent invitation letters by mail, fax and/or email, each of which include participant information and an informed consent form for the practice owner. Practices will then be contacted by phone to provide additional study information. As soon as the practice owner has given written consent, a visit by a study nurse from the Center for Clinical Trials in Essen (in German, *ZKSE, Zentrum für klinische Studien Essen*), is scheduled. During this visit, the study nurse will obtain informed consent from each participating team member, distribute and collect the baseline questionnaire data, and complete an observational occupational health and safety form. In the consent form, participants are informed that they are free to discontinue study participation at any time. Questionnaires will be checked for completeness of data. To promote participant retention and to prevent loss to follow up, each participant will receive 50€ after the intervention. In addition, IMPROVE*job* facilitators will motivate participants to complete the study.

### Inclusion and exclusion criteria

Practices will be included if at least one practice physician is registered as a GP with the Association of Statutory Health Insurance Physicians of North Rhine with or without teaching obligations. Practice teams are eligible for participation if at least one physician with leadership responsibilities and at least one practice assistant provide informed consent for study participation. Physicians with leadership responsibilities include physician practice owners and employed practice physicians with respective duties. Our aim is to recruit all members of a practice team including employed physicians and practice assistants in training.

Exclusion criteria are a foreseeable special situation that might interfere with the completion of the study, such as practice relocation or retirement of the practice owner; practices that are not primarily involved in primary care (e.g. predominantly providing psychotherapy or pain therapy); and practices that participated in the development of the IMPROVE*job* intervention or in the feasibility study of the intervention.

### Randomisation

General practices will be randomised by means of several random lists. These lists include single and group practices and teaching and non-teaching practices, to achieve a roughly equal distribution in both the intervention and the control groups. We chose the stratification factor of (a) single/group practice, because structural characteristic, leadership responsibilities and indicators of psychological wellbeing may differ between practice types [10, 16] and (b) teaching/non-teaching practice, as this factor may influence the motivation to participate in the intervention (and may indirectly influence its effects). The randomisation list is generated by an employee of the ZKSE who is not involved in the selection of the practices. Practices will be randomised after baseline data collection, to ensure that study nurses are blinded for the first site visit. The randomisation allocation will be transferred to scientists at the Institute of General Practice and Family Medicine in written form, who will inform the practices by phone and letter. Blinding of the scientists and the practice personnel is not possible due to the participatory concept of the intervention, which includes workshops. Data analysts will follow a predefined protocol for analysis to avoid bias.

### Questionnaires and outcome measures

The primary outcome of this study is an improvement in job satisfaction. To measure job satisfaction, the respective scale of the German version of the Copenhagen Psychosocial Questionnaire based on the international COPSOQ III version (German COPSOQ, version 2018) will be used (www.COPSOQ.de). The COPSOQ is a validated questionnaire that measures psychosocial factors at work [26–28]. The job satisfaction scale of this instrument consists of 6 items (B11: 1–5) and is combined with an additional global item (B11: 7 “how pleased are you with your job as a whole, everything taken into consideration?”). The outcome “ob satisfaction” was selected as the primary outcome, because the IMPROVE*job* study evaluates a complex intervention, which addresses various factors known to influence the psychological wellbeing of practice personnel.

The following additional COPSOQ scales will be used as secondary outcomes: quantitative demands (B1: 1, 3, 5), emotional demands (B1: 6–7), work pace (B1: 2, 4), work-privacy conflict (B2: 1–2), and additional items (B2: 3–4, B2: 5) “delimitation” (B2: 6–7), predictability (B6: 1–2), role clarity (B6: 3–5), role conflicts (B6: 6–8); social support (B8: 1–4), feedback (B8: 5–6), social relations (B8: 7), sense of community (B8: 8–9) and bullying (B8: 10). To calculate scale scores for each dimension, the COPSOQ will be transformed as recommended [29]. Scales will be transformed into scores ranging from 0 (minimum value, “do not agree at all”) to 100 points (maximum value, “fully agree”).

The following additional aspects will be addressed using questionnaires: (1) leadership, (2) general health (3) work behaviour, (4) chronic stress, (5) occupational health and safety culture, (6) stress coping strategies applied by participants, (7) work organisational issues including waiting times, team roles and team activities and (8) team activities and roles.

#### Leadership

Leadership is assessed with items from the questionnaires on Integrative Leadership (FIF, *Fragebogen zur Integrativen Führung*) and Leader-Member-Exchange (LMX-7).

The FIF questionnaire [30, 31] focuses on six dimensions of transformational leadership (fostering innovation, team spirit development, performance development, individuality focus, providing a vision, being a role model), two dimensions of transactional leadership (goal setting, management by exception) (module a: items 1–32) [32] and on laissez-faire and destructive leadership (module d: items 65–68) [33]. All items are answered on a 5-point Likert scale. Physicians with leadership responsibilities will assess themselves, whereas other practice physicians and practice assistants will evaluate their leaders. The instrument allows for a comparison of answers provided by leaders and other personnel.

The LMX-7 measures the relationship quality between employees and supervisors (leaders) [34, 35]. It consists of 7 items with 5-point Likert scales. The LMX values are added to a “sum-score. A high score reflects a positive assessment of the quality of the relationship with the supervisor. Again, practice owners will assess themselves, while employed practice physicians and practice assistants assess their leaders:

1. General health is assessed using a brief burnout assessment, the World Health Organization-Five Well-Being Index (WHO-5) and the Work Ability Index (WAI).
  Burnout is measured using two items from the Maslach Burnout Inventory (emotional exhaustion: “I feel burned out from my work”, depersonalization: “I have become more callous toward people since I took this job”) [36]. This brief measure of burnout was shown to provide sufficient information on the likelihood of high burnout among physicians and medical students [37, 38]. The score ranges from 0 = never to 6 = every day, and the raw values are processed to obtain a score for emotional exhaustion and depersonalization, which can be compared to the results obtained by the full Maslach Burnout Inventory [38].
  Current well-being during the last – 14 days is assessed using the WHO-5 (1998 version) [34, 39]. It consists of 5 items with 6-point Likert scales (5 = all of the time to 0 = at no time). The scores are added to a sum-score ranging from 0 to 25 and are then multiplied by 4 to give the final score, with 0 denoting the worst and 100 representing the best imaginable wellbeing [34].
  Work ability is measured using a single item of the WAI: “personal prognosis of work ability two years from now” on a 3-point Likert scale [40, 41].
2. Work behavior is assessed using short versions of the Occupational Self-Efficacy Scale (BSW, *Berufliche Selbstwirksamkeit*) and the Work-related Behaviour and Experience Patterns questionnaire (AVEM-44).
  Self-efficacy is measured using a short version of the BSW [42]. The instrument consists of 8 items with 6-point Likert scales.
  The interplay between work, personality and health is assessed using the short version of the AVEM-44 [43–46]. The AVEM addresses three aspects, which are particularly important for coping with occupational challenges. It consists of 44 items across three domains: professional commitment (20 items across five scales: subjective importance of work; professional ambition; willingness to work to exhaustion; striving for perfection; distancing ability); coping capacity (12 items across three scales: tendency to resign in the face of failure; proactive problem-solving; inner calm and balance); subjective wellbeing (12 items across three scales, experience of success at work; satisfaction with life; experience of social support). Each scale consists of four items, which are measured on a 5-point Likert scale. AVEM identifies four patterns which describe coping strategies for occupational stress: healthy-ambitious (pattern G), unambitious (pattern S), excessively ambitious (risk pattern A) and burnout (risk pattern B).
3. Chronic stress is measured using the German short version of the Screening Scale of the Trier Inventory for the Assessment of Chronic Stress (TICS-SSCS) [47, 48]. This instrument retrospectively measures strain due to chronic stress, for 3 months. It consists of 12 items on 5-point Likert scales (0 = “never” and 4 = “very often”). The TICS-SSCS values are added to a sum-score. The score ranges from 0 to 48 with 0 denoting “never stressed” and 48 “very often stressed”, and reflects subjective strain due to chronic stress.
4. Occupational health and safety is measured using questions from previous studies [49–52], physicians with leadership responsibilities will answer 29 items on occupational safety culture as reported by the practice owners; other staff will answer 28 items.
5. Individual coping strategies are assessed using 26 items specifying various option for stress prevention, e.g. playing an instrument, going for a walk or hiking outdoors and support from friends. Items were derived from previous studies [10].
6. Various issues relating to work organisation will be addressed, e.g. estimated duration and peaks of waiting times for private and non-private patients, working overtime due to problems with organisational workflows, reasons for waiting times.
7. Team activities and roles will be addressed by requesting the frequencies of team sessions and other team activities. Also participants are asked to self-assess their typical personal team role from a choice of nine options.

**Fig. 2:**
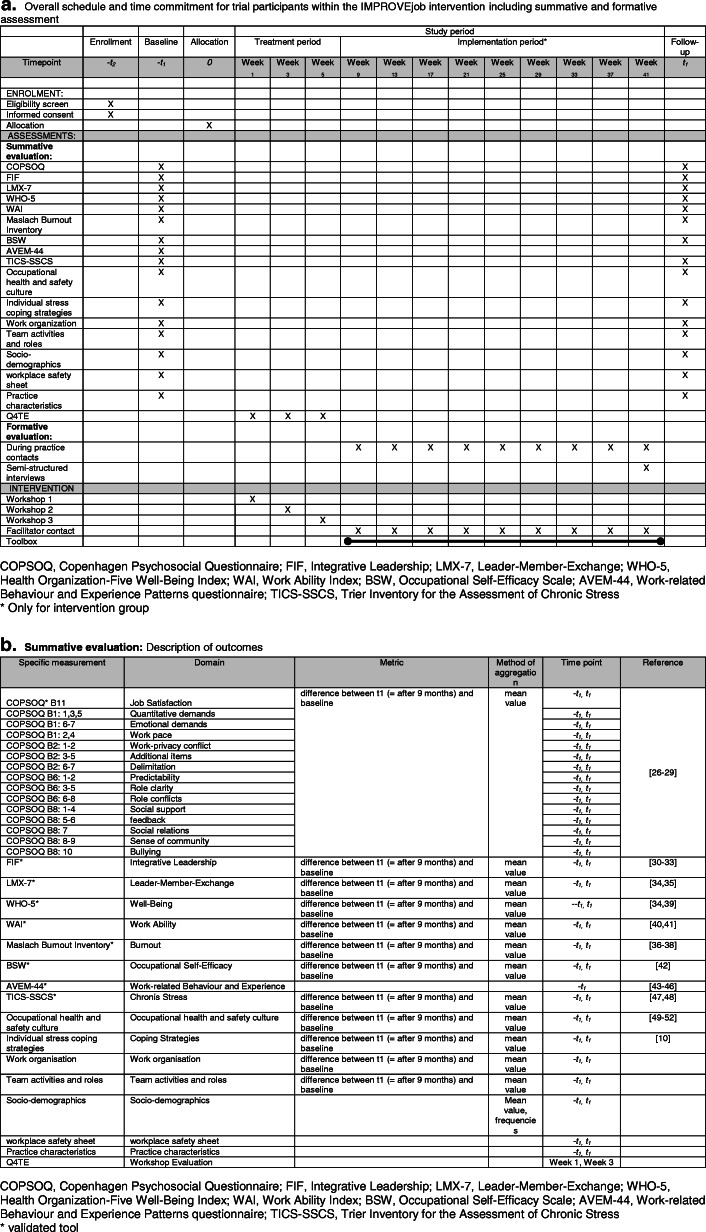
**a** Overall schedule and time commitment for trial participants within the IMPROVEjob intervention. b Summative evaluation: description of outcomes. COPSOQ, Copenhagen Psychosocial Questionnaire; FIF, Integrative Leadership; LMX-7, Leader-Member-Exchange; WHO-5, Health Organization-Five Well-Being Index; WAI, Work Ability Index; BSW, Occupational Self-Efficacy Scale; AVEM-44, Work-related Behaviour and Experience Patterns questionnaire; TICS-SSCS, Trier Inventory for the Assessment of Chronic Stress

Socio-demographic characteristics and practice characteristics will be collected as moderating variables. Stable co-variables (e.g. socio-demographic characteristics of study participants, work-related experience and behavioural patterns (AVEM) [53], practice characteristics) will be collected only at baseline, while changeable variables will be measured at both at baseline and follow up. Study nurses will offer an optional workplace safety sheet at baseline, to be completed together with the practice leader. The sheet addresses issues such as hygiene, emergency exits and skin protection instructions. Participation is voluntary for the leaders; a copy of the report will be provided immediately. For an overall schedule see Fig. 2a.

### Intervention

The multimodal participatory intervention was initiated by a general practice physician and researcher (BMW). The intervention was designed cooperatively by researchers from the fields of general practice and family medicine and those from occupational medicine, psychosomatic medicine, operations practice research and workplace health promotion. The intervention focuses on the areas of leadership, work processes and work organization, communication, occupational health and safety and workplace health promotion. It is composed of three elements.

#### IMPROVE*job* workshops

The intervention begins with a series of three workshops each lasting 4 h. The three workshops will be held within 1 month with 2-week intervals in between. About 5–6 practices will take part in each workshop depending on the number of staff in each practice. This allows for about 8–10 physicians and 15–25 practice assistants per workshop series. All workshops include presentations by the research team and interactive elements with self-reflection and peer group exchanges.

The specific contents of the workshop are as follows:

1. Workshop 1 for physicians with leadership responsibilities (practice owners and employed physicians with leadership responsibilities): this executive workshop addresses the topics “role of the executive”, “leadership styles” and “relational leadership competence” including “transactional and transformational leadership”. The workshop includes theoretical inputs delivered via visual presentation, small group interactions, and a skills training session. The later training is designed to allow for training in leadership aspects in a simulated practice scenario with a simulated practice assistant: one participant is asked to take on the role of a physician leader who is facing a conflict between team members with leadership responsibilities. The workshop offers leaders the opportunity to reflect on their own leadership role and values, to increase the awareness for and impact of stress prevention for all team members, and to learn different leadership styles. In particular, different aspects of the relational, transactional and transformational leadership approaches and their effects on employees´ health and team-building are addressed.
2. Workshop 2 for physicians with leadership responsibilities and all practice employees: This workshop addresses issues relating to the communication between practice personnel and patients. Using a theoretical framework and interactive skills sessions with trained simulation patients, participants will learn how to analyse encounters with patients that they perceive as challenging and how to better communicate in such situations. Training in challenging situations is provided by drawing on typical scenarios in primary care practices.
3. Workshop 3 for physicians with leadership responsibilities and all practice employees: this workshop addresses issues relating to “work organisation including appointment scheduling”, “occupational health and safety” and “workplace health promotion”. At the end of the workshop series, practices will be empowered and supported with implementation aids such that they are able to adjust their daily routines according to self-selected goals. Practices will be encouraged to set targets for their implementation phase.

#### IMPROVE*job* toolbox

The toolbox includes materials presented in the workshops and supplemental material. It comprises two booklets (one for physicians with leadership responsibilities and one for practice employees), a desk calendar and a personal code allowing access to a secured web space with additional material for downloading.

- The “management logbook” (for physicians with leadership responsibilities): this folder contains material for workshops 1–3 plus supplements.
- The “employee logbook” provides materials for workshops 2 and 3 plus supplemental material.
- The desk calendar for practice teams presents a variety of issues from the workshops in a concise card format, e.g. advice on communication with so-called “difficult patients”, on occupational health and safety, and on issues of individual health promotion such as relaxation and ergonomics.
- Supplemental material for download, e.g. practice checklists and other tools, will be provided via a secured webspace. The personalised access allows for the evaluation of webspace use on an individual level.

#### IMPROVE*job* facilitators

The facilitators are trained members of the research team who will accompany the practices during the change processes by on-site meetings in the practice and/or by phone. Prior to the workshops, the facilitators will be trained in change processes, the setting of GP practices and qualitative data collection in two training sessions. The facilitators concentrate on processes and developments within the practices, but they do not engage in active coaching. They are conceptualised as process companions who remind the practice teams of the project and support the implementation of the intervention. In addition, the facilitators will collect qualitative data in the practices to analyse the relevance, feasibility and implementation of the intervention. The facilitators will take part in the intervention workshops where they will meet the study participants. Also, they will note the practice goals set by each participating practice. After the workshops the facilitators will offer practices to contact them regularly, e.g. once a month, to facilitate the implementation.

### Control group

This study will use a waiting-list control approach. After completion of the follow-up data collection, practices in the control group will be offered the same IMPROVE*job* workshops as practices in the intervention group, including access to the toolbox. The project duration and funding does not allow for IMPROVE*job* facilitators to be involved in the waiting list control group.

### Sample size calculation

The primary hypothesis of this study is that the IMPROVE*job* intervention will have an effect on job satisfaction among personnel working in general practices. The null and alternative hypotheses to be tested are:

$$ {\mathrm{H}}_0:{\upmu}_{\mathrm{I}}={\upmu}_{\mathrm{C}} $$

$$ {\mathrm{H}}_{\mathrm{A}}:{\upmu}_{\mathrm{I}}\ne {\upmu}_{\mathrm{C}}, $$

where μ_I_ and μ_C_ denote the mean score difference on the COPSOQ job satisfaction scale between baseline and after 9 months, in the intervention arm and in the control arm.

We aim for power of (at least) 80% with a two-sided significance level α = 0.05. The calculations will be carried out by means of the clustered *t* test [54, 55], assuming an intra-cluster correlation coefficient (ICC) of 0.05 [56]. All power analyses will be made using the software PASS 13, option “Tests for Two Means in a Cluster-Randomized Design”.

Assuming a mean value plus/minus standard deviation for the primary endpoint of 73.6 ± 15 points [57], we calculate that a sample size of 52 clusters (26 practices per arm), each with 3 participating staff members per practice, will be sufficient to detect a change of 10% (7.3 points) with power of 81% at the chosen significance level. With 4 participants per practice, the power will increase to 89%.

Conversely, with 52 practices and 4 participants per practice, the study would have power of 80% to detect a change of 0.43 SD in the primary endpoint (and 0.39 SD with 5 participants per practice). This is comparable to the “minimally important difference” (MID) of 0.4 SD proposed as a relevant change in the COPSOQ job satisfaction score [58]. As the values and standard deviation are lower on this scale for physicians than for practice assistants [57, 59], the sample size calculation is rather conservative. Assuming two practice drop-outs in each of the two groups (intervention and control), we will include physicians and practice assistants from 56 general practices in the study.

### Data management, statistical analyses and steering committee

Data management will be carried out by the ZKSE according to standardised procedures as defined in current standard operating procedures (SOPs). The data management system used by the ZKSE has an integrated audit trail and is Good Clinical Practice (GCP)-compliant. Data will be entered by appropriately trained data-entry staff who are familiar with the study specifics. Double data-entry will be used to ensure data quality. Issues of missing data will be addressed by imputation methods according to standard [60]. All personal data will be kept confidential in an access-restricted database. All analyses will be performed using pseudonymised data. The pseudonymised data will be stored at the ZKSE and the Institute of General Practice and Family Medicine of the University of Bonn. The latter institute will manage access to the data set.

Regarding the primary outcome, we hypothesise that the participatory IMPROVE*job* intervention will improve job satisfaction among practice personnel. A before-and-after comparison between the intervention and the control group will be used for the evaluation. To control for response shift with respect to job satisfaction, a “then-test” will be applied using a separate sheet to be filled after collection of the follow-up questionnaires [61]. For the statistical analysis, the COPSOQ scales will be transformed according to instrument-specific algorithms [27]. The confirmatory analysis to measure the effect of the intervention will be carried out as an intention-to-treat analysis, i.e. all participants who completed the job satisfaction scale at baseline and follow up will be included. The primary analysis will be conducted using a linear mixed model adjusted for clustering and the randomisation strata. Per protocol, the analysis will be performed with the data on physicians with leadership responsibility who took part in the management workshop and in at least one of the two team workshops, and with data on practice assistants who took part in at least one team workshop. Additional analyses will focus on secondary outcomes and on associations between various independent and dependent variables. All analyses will respect the cluster-randomised nature of the study design including approaches for analysis of team aspects within the practices. All measures of the summative evaluation will compare mean differences between follow up and baseline (Fig. 2b).

The project is supported by a steering committee with scientists from various fields (general practice and family medicine, occupational psychology, sociology) and representatives of organisations relevant for transfer and dissemination (statutory health insurance, regional medical association, association for health service and welfare work, regional chamber of industry and commerce).

## Discussion

The participatory IMPROVE*job* intervention is a novel approach aimed at addressing typical work-related issues encountered in general practices. It is designed to empower teams to analyse their situation and apply self-selected strategies to modify daily work routines. In contrast to many stress-reducing intervention studies [62], our approach integrates behavioural and environmental changes.

Regarding didactics, the IMPROVE*job* study applies modern learning strategies such as interactive sessions, peer learning and skills training in leadership and patient-staff communication [63]. The latter allows for a simulation of real-life scenarios and training close to reality following concepts of an enactive mastery experience [64]. The latter was recently shown to be effective in training residents for in-house leadership [65] but has not been evaluated in leadership training for physicians with leadership responsibilities or practice assistants in the outpatient setting.

Given the high prevalence of psychological strain, sick leave due to depression and respective early retirement in various job domains in Germany, the German Federal Ministry of Education and Research has setup the Funding Initiative “Healthy – for life” (in German, *Gesund – ein Leben lang*). The IMPROVE*job* study is funded within this initiative. Respecting the described societal context, the IMPROVE*job* intervention will be evaluated in general practices, which are considered to be models for small and medium sized enterprises, yet - if proven effective - the intervention will be disseminated in other medical settings and evaluated for its transferability to other occupational fields.

## Trial status

This is protocol version #1. The trial is ongoing. The recruitment of participants started on 5 September 2019 and was completed on 4 March 2020.

## Data Availability

There are no plans to grant access to full protocol, participant-level dataset or statistical code.

## Abbreviations

AVEM-44: Work-related Behaviour and Experience Patterns questionnaire
BSW: Occupational Self-Efficacy Scale (*Berufliche Selbstwirksamkeit*)
COPSOQ: Copenhagen Psychosocial Questionnaire
FIF: Questionnaires on Integrative Leadership (*Fragebogen zur Integrativen Führung*)
GP: General practitioner
LMX-7: Leader-Member-Exchange
TICS-SSCS: Screening Scale of the Trier Inventory for the Assessment of Chronic Stress
WAI: Work Ability Index
WHO: World Health Organisation
WHO-5: World Health Organization-5 Well-Being Index
