# Correction to: IMPROVEjob – Participatory intervention to improve job satisfaction of general practice teams: a model for structural and behavioural prevention in small and medium-sized enterprises – a study protocol of a cluster-randomised controlled trial

**DOI:** 10.1186/s13063-020-04914-x

**Published:** 2020-12-08

**Authors:** Birgitta M. Weltermann, Christine Kersting, Claudia Pieper, Tanja Seifried-Dübon, Annegret Dreher, Karen Linden, Esther Rind, Claudia Ose, Karl-Heinz Jöckel, Florian Junne, Brigitte Werners, Verena Schroeder, Jean-Marie Bois, Achim Siegel, Anika Thielmann, Monika A. Rieger, Stefanie Kasten, on behalf of the IMPROVEjob consortium, M. A. Rieger, E. Rind, A. Siegel, A. Wagner, E. Tsarouha, B. Weltermann, S. Kasten, K. Linden, L. Degen, A. Thielmann, F. Junne, T. Seifried-Dübon, A. Hermann-Werners, F. Stuber, S. Zipfel, B. Werners, M. Grot, K-H Jöckel, C. Pieper, V. Schröder, J-M Bois, A-L Eilerts, M. Brinkmann, C. Kersting, S. Emerich, S. Burgess, M. Hippler, A. Dreher, C. Ose, L. Koppka, J. Block

**Affiliations:** 1 Institute of General Practice and Family Medicine, University Hospital Bonn, University of Bonn, Venusberg-Campus 1, 53127 Bonn, Germany; 2 Institute for General Medicine, University Hospital Essen, University of Duisburg-Essen, Hufelandstr 55, 45122 Essen, Germany; 3 Institute for Medical Informatics, Biometry and Epidemiology, University Hospital Essen, University of Duisburg-Essen, Hufelandstr 55, 45147 Essen, Germany; 4 Department of Psychosomatic Medicine and Psychotherapy, University Hospital Tuebingen, Osianderstraße 5, 72076 Tuebingen, Germany; 5 Institute of Occupational and Social Medicine and Health Services Research, University Hospital Tuebingen, Wilhelmstr 27, 72074 Tuebingen, Germany; 6 Center for Clinical Trials, University Hospital Essen, University of Duisburg-Essen, Hufelandstr. 55, 45147 Essen, Germany; 7 Institute for Operations Research, Ruhr University Bochum, Universitätsstr 150, 44801 Bochum, Germany

**Correction to: Trials 21, 532 (2020)**

**https://doi.org/10.1186/s13063-020-04427-7**

After publication of the original article [1], the authors have notified us that their originally submitted Fig. 1 was mistakenly replaced with an incorrect figure during editing.

- Originally published Fig. 1:

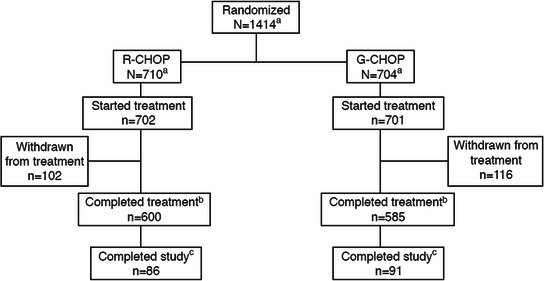

- Correct Fig. 1:

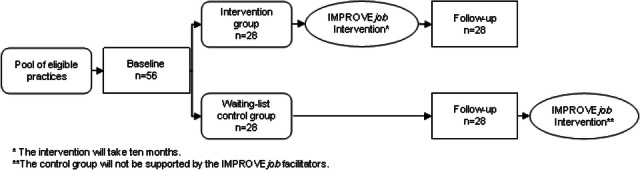

The Publisher apologizes for this error and any confusion caused.

The original article has also been updated.
